# Serum Ratio of Free Triiodothyronine to Thyroid-Stimulating Hormone: A Novel Index for Distinguishing Graves’ Disease From Autoimmune Thyroiditis

**DOI:** 10.3389/fendo.2020.620407

**Published:** 2021-01-08

**Authors:** Zhiyong Wu, Yu Zhu, Min Zhang, Chen Wang, Lingli Zhou, Wei Liu, Wenjia Yang, Meng Li, Simin Zhang, Qian Ren, Xueyao Han, Linong Ji

**Affiliations:** Departments of Endocrinology and Metabolism, Peking University People’s Hospital, Peking University Diabetes Center, Beijing, China

**Keywords:** Graves’ disease, autoimmune thyroiditis, thyrotoxicosis, thyroid function test, differential diagnosis

## Abstract

**Objective:**

Graves’ disease (GD) and autoimmune thyroiditis (AIT) are two major causes of thyrotoxicosis that require correct diagnosis to plan appropriate treatment. The objectives of this study were to evaluate the usefulness of thyroid-related parameters for distinguishing GD from AIT and identify a novel index for differential diagnosis of thyrotoxicosis.

**Design:**

This retrospective study was performed using electronic medical records in Peking University People’s Hospital (Beijing, China).

**Methods:**

In total, 650 patients with GD and 155 patients with AIT from December 2015 to October 2019 were included in cohort 1. Furthermore, 133 patients with GD and 14 patients with AIT from December 2019 to August 2020 were included in cohort 2 for validation of the novel index identified in cohort 1. All patients were of Chinese ethnicity and were newly diagnosed with either GD or AIT. Thyroid-related clinical information was collected before intervention by reviewing the patients’ electronic medical records. Receiver operating characteristic curve analysis was used to identify the optimal cutoff for distinguishing GD from AIT.

**Results:**

In cohort 1, thyroid-stimulating hormone (TSH) receptor antibody was identified as the best indicator for distinguishing GD from AIT. The area under the receiver operating characteristic curve was 0.99(95% confidence interval: 0.98–0.99, p<0.0001)and the optimal cutoff was 0.84 IU/l (98% sensitivity and 99% specificity). The free triiodothyronine (FT3)/TSH ratio (FT3/TSH) was the second –best for distinguishing GD from AIT, the area under the receiver operating characteristic curve of FT3/TSH was 0.86 (95% confidence interval: 0.84–0.88, p<0.0001); its optimal cutoff was 1.99 pmol/mIU (79% sensitivity and 80% specificity). Its effectiveness was confirmed in cohort 2 (81% sensitivity and 100% specificity).

**Conclusions:**

The FT3/TSH ratio is a new useful index for differential diagnosis of thyrotoxicosis, especially when combined with TRAb.

## Introduction

Thyrotoxicosis is a common endocrine syndrome characterized by excess thyroid hormone action at the tissue level and disproportionately high circulating thyroid hormone concentrations. This disorder has two major etiologies: 1. excess thyroid hormone synthesis and secretion by the thyroid gland due to Graves’ disease (GD), toxic multinodular goiter, or toxic adenoma (1–3); and 2. release of thyroid hormones due to destruction of thyroid tissue caused by thyroiditis (e.g., in patients with subacute thyroiditis, Hashimoto’s thyroiditis, painless thyroiditis, or postpartum thyroiditis) (4–6).

GD and autoimmune thyroiditis (AIT; includes Hashimoto’s thyroiditis, painless thyroiditis and postpartum thyroiditis) are two of the most frequent etiologies of thyrotoxicosis. Because some patients with GD lack the typical clinical manifestations (e.g., goiter, ophthalmopathy, and thyroid-stimulating hormone [TSH] receptor antibody [TRAb] positivity) (7, 8), it can be difficult to distinguish GD from AIT. However, this determination is critical because of the considerable differences in treatment regimens. Patients with GD receive either antithyroid drug treatment, radioactive iodine (RAI), or surgery; patients with AIT exhibit self-limiting disease that requires follow-up alone (9).

Although the RAI uptake test is a useful method for distinguishing thyrotoxicosis etiologies, it is inappropriate for patients who are pregnant, lactating or exposed to excess iodine. Thus, other methods have been used for diagnosis of thyrotoxicosis etiology, including the ratio of total triiodothyronine (TT3) to total thyroxine (TT4) (10–13), the TRAb level (14, 15), and the ratio of free triiodothyronine (FT3) to free thyroxine (FT4) (16–19); however, TT3/TT4 and FT3/FT4 ratios remain controversial because of poor replication in larger studies and limited sensitivity/specificity. Moreover, a precise cutoff for the TRAb level has not been established. Accordingly, new and better methods for distinguishing thyrotoxicosis etiologies are necessary in clinical practice, especially in limited-resource settings. In the current study, we evaluated the usefulness of FT3, FT4, and TSH levels, as well as FT3/FT4 and TT3/TT4 ratios, in distinguishing GD from AIT. We also endeavored to identify a novel index for diagnosis of thyrotoxicosis etiology.

## Materials and Methods

### Study Design and Ethical Approval

This retrospective study used data from hospital electronic medical records. The study was conducted in compliance with the tenets of the Declaration of Helsinki and the study protocol was approved by the Ethics Committee of Peking University People’s Hospital (Beijing, China).

### Study Populations

#### Cohort 1: Evaluation of Thyroid-Related Parameters for Distinguishing GD From AIT

In total, 805 Chinese outpatients with newly diagnosed autoimmune-related thyrotoxicosis were included in this portion of the study: 650 patients with GD and 155 patients with AIT. All patients had visited the Endocrinology and Metabolism Department of People’s Hospital Peking University in Beijing from December 2015 to October 2019. Patients were diagnosed with thyrotoxicosis if they met the following criteria: 1) presence of signs and symptoms of thyrotoxicosis; 2) detection of diffuse thyroid lesions during physical examination or color Doppler ultrasound; 3) presence of low serum TSH levels and elevated serum FT3 and/or FT4 levels; 4) exclusion of other causes of thyrotoxicosis (e.g., pregnancy, toxic multinodular goiter, toxic adenoma, amiodarone-induced thyrotoxicosis, and/or exogenous thyroxine intake); 5) absence of antithyroid drug treatment; 6) absence of fever, elevated erythrocyte sedimentation rate, or any diseases/medications that might influence measurements of thyroid function; 7)regardless of serum TRAb levels.

If patients with thyrotoxicosis had high RAI or technetium-99m (Tc-99m) uptake, or if they required antithyroid drug treatment to maintain normal thyroid hormone levels, they were diagnosed with GD, regardless of the presence or absence of Graves’ ophthalmopathy, pretibial myxedema and/or goiter. If patients with thyrotoxicosis had low RAI or Tc-99m uptake in thyroid scintigraphy, or if they exhibited spontaneous hypothyroidism without any antithyroid drugs treatment, they were diagnosed as AIT. Because high iodine intake could cause low rate of RAI uptake, some patients with GD might have been misdiagnosed with AIT in this condition. To avoid this error, only the patients without intake of food and drugs containing iodine within the prior 2-4 weeks were determined for RAI uptake rate.

### Cohort 2: Validation of Novel Index for Distinguishing GD From AIT

In total, 147 patients with newly diagnosed thyrotoxicosis were included in this portion of the study: 133 patients with GD and 14 patients with AIT. All patients had visited the Endocrinology and Metabolism Department of People’s Hospital Peking University in Beijing from December 2019 to August 2020. Data from these patients were used to validate the novel index for distinguishing GD from AIT (identified in cohort 1). The inclusion and exclusion criteria were identical to those in cohort 1.

### Clinical and Laboratory Assessments

Pre-intervention thyroid function and autoantibody test results were collected from patients’ electronic medical records in Peking University People’s Hospital. All patients underwent examinations of FT3, FT4, and TSH. Some patients underwent assessments of RAI or Tc-99m uptake; for patients without those assessments, follow-up information was reviewed to determine their definitive diagnosis.

Serum levels of TT3, TT4, FT3, FT4, TSH, TRAb, thyroid peroxidase antibody (TPO-Ab), and thyroglobulin antibody (TgAb) were determined using automated chemiluminescent immunoassays (ADVIA centaur XP; Siemens). The reference ranges provided by the manufacturer used in this study were as follows: TSH, 0.55–4.78 µIU/ml; FT4, 11.45–23.17 pmol/L; FT3, 3.5–6.5 pmol/L; TT4, 41.18–162.16 nmol/L(3.2–12.6 µg/dl); TT3, 0.92–2.77 nmol/L(60–180 ng/dl; Tg–Ab, 0–60 IU/L; and TPO-Ab, 0–60 IU/L. The upper detection limits were as follows: FT4, 154.8 pmol/L; FT3, 30.8 pmol/L; TT4, 386.10 nmol/L(30 μg/dl; TT3, 12.32 nmol/L(800 ng/dl; Tg-Ab, 500 IU/L; and TPO-Ab, 1,300 IU/L; and the lower detection limit for TSH was 0.001 µIU/ml; TRAb levels were measured by electrochemiluminescence immunoassays (Cobas e601; Roche Diagnostics). The lower and upper detection limits for TRAb were 0.3 IU/L and 40 IU/L, respectively.

### Statistical Analysis

Continuous variables were presented as means ± standard deviations if the data were normally distributed, or as medians (interquartile range) if the data were not normally distributed. Categorical variables were presented as numbers and percentages. Variables without normal distributions were subjected to natural logarithm transformation to obtain a normal distribution prior to statistical analysis. Student’s t-test or the Mann-Whitney U test were used to compare clinical characteristics between patients with GD and those with AID. Receiver operation characteristic (ROC) curve analysis was performed to identify optimal cutoff values for GD. Sensitivity and specificity values were estimated from ROC curves. All statistical tests were performed using SPSS Statistics version 22.0 for Mac (IBM Corp., Armonk, NY, USA) or MedCalc version 19 (Ostend Belgium). *P* < 0.05 was considered statistically significant.

## Results

### Clinical Characteristics of Patients With GD and Those With AIT

As shown in **Tables 1** and **2**, thyrotoxicosis was more severe in patients with GD than in patients with AIT. This trend was consistent among patients in both cohort 1 and 2. The serum levels of TT3, TT4, FT3, FT4, TPO-Ab, and TRAb were significantly higher in patients with GD than in patients with AIT, while the serum level of TSH was significantly lower in patients with GD than in patients with AIT. Moreover, the ratios of TT3/TT4, FT3/FT4, FT3/TSH, FT4/TSH, TT3/TSH, and TT4/TSH were significantly higher in patients with GD than in patients with AIT.

**Table 1:** Clinical characteristics of patients with Graves’ disease and those with autoimmune thyroiditis in Cohort 1.

Items	GD (n = 650)	AIT (n = 155)	P
**Age (yrs)**	41 ± 13	39 ± 13	0.109
**Sex (Female, %)**	517 (79.5%)	115 (74.2%)	0.157
**FT3 (pmol/L)**	18.52 (12.31, 29.34)	9.86 (7.63, 12.72)	<0.001
**FT4 (pmol/L)**	47.91 (33.85, 60.08)	32.20 (26.94, 41.76)	<0.001
**FT3/FT4**	0.37 ± 0.11	0.33 ± 0.11	<0.001
**TT3 (ng/dl )**	341.19 (240.67, 476.48)	206.88 (168.09, 256.05)	<0.001
**TT4 (ug/dl)**	17.80 (13.90, 22.70)	14.10 (11.60, 17.05)	<0.001
**TT3/TSH (ug/uIU)**	0.84 (0.42, 1.78)	0.20 (0.09, 0.37)	<0.001
**TT4/TSH (ug/uIU)**	42.63 (23.02, 81.91)	14.56 (6.02, 24.58)	<0.001
**TT3/TT4 (ng/ug)**	20.13 ± 5.05	16.00 ± 6.10	<0.001
**TSH (uIU/ml)**	0.004 (0.002, 0.007)	0.011 (0.007, 0.021)	<0.001
**FT3/TSH (pmol/mIU)**	4.49 (2.21, 8.86)	0.93 (0.46, 1.80)	<0.001
**FT4/TSH (pmol/mIU)**	12.03 (6.220, 25.77)	3.10 (1.39, 5.90)	<0.001
**Tg-Ab (IU/ml)**	142.30 (34.75, 316.05)	196.10 (56.075, 306.425)	0.193
**TPO-Ab (IU/ml)**	543.90 (53.90, 1300.00)	44.10 (28.00, 1300.00)	<0.001
**TRAb (IU/L)**	7.48 (3.67, 16.35)	0.30 (0.30, 0.31)	<0.001

GD, Graves’ disease; AIT, autoimmune thyroiditis; TRAb, TSH receptor antibody; TT3, total triiodothyronine; TT4, total thyroxine; FT3, free triiodothyronine; FT4, free thyroxine; TSH, thyroid stimulating hormone; TPO-Ab, thyroid peroxidase antibody; Tg-Ab, thyroglobulin antibody.

**Table 2:** Clinical characteristics of patients with Graves’ disease and those with autoimmune thyroiditis in Cohort 2.

Items	GD (n = 133)	AIT (n = 14)	P
**Age (yrs)**	39.73 ± 13.61	33.07 ± 11.60	0.080
**Sex (Female, %)**	104 (78.2%)	12 (85.7%)	0.735
**FT3 (pmol/L)**	20.81 (13.41, 30.80)	9.06 (7.44, 12.14)	<0.001
**FT4 (pmol/L)**	52.90 (39.36, 75.93)	29.32 (25.56, 38.59)	<0.001
**FT3/FT4**	0.35 ± 0.09	0.30 ± 0.05	0.003
**TT3 (ng/dl )**	363.27 (260.92, 486.18)	204.42 (158.30, 259.70)	<0.001
**TT4 (ug/dl)**	19.30 (14.65, 24.30)	13.20 (11.30, 16.05)	<0.001
**TT3/TSH (ug/uIU)**	0.68 (0.43, 1.31)	0.13 (0.04, 0.26)	<0.001
**TT4/TSH (ug/uIU)**	36.63 (24.57, 61.00)	7.81 (2.84, 16.66)	<0.001
**TT3/TT4 (ng/ug)**	19.67 ± 4.45	15.34 ± 2.59	0.001
**TSH (uIU/ml)**	0.005 (0.003, 0.007)	0.016 (0.093, 0.420)	<0.001
**FT3/TSH (pmol/mIU)**	4.06 (2.62, 7.08)	0.69 (0.18, 1.22)	<0.001
**FT4/TSH (pmol/mIU)**	10.90 (7.02, 24.75)	2.07 (0.61, 4.57)	<0.001
**Tg-Ab (IU/ml)**	157.65 (47.58, 318.85)	109.70 (18.18, 159.05)	0.126
**TPO-Ab (IU/ml)**	1300 (102.88, 1300)	31.05 (28.00, 1300)	0.008
**TRAb (IU/L)**	7.49 (3.45, 16.79)	0.31 (0.30, 0.73)	<0.001

GD, Graves’ disease; AIT, autoimmune thyroiditis; TRAb, TSH receptor antibody; TT3, total triiodothyronine; TT4, total thyroxine; FT3, free triiodothyronine; FT4, free thyroxine; TSH, thyroid stimulating hormone; TPO-Ab, thyroid peroxidase antibody; Tg-Ab, thyroglobulin antibody.

### Evaluation of Thyroid-Related Parameters for Distinguishing GD From AIT Among Patients in Cohort 1

As shown in **Table 3**, the areas under the ROC curve (AUC) for TSH and TRAb levels, as well as the TT3/TSH, TT4/TSH, FT3/TSH, and FT4/TSH ratios, were all >0.8. The AUC of TRAb was greater than the AUCs of TSH, TT4/TSH, TT3/TSH, FT3/TSH, and FT4/TSH (all p<0.0001). The AUC of FT3/TSH was greater than the AUCs of FT4/TSH, TT4/TSH, and TSH(all p<0.0001); it was also greater than the AUC of TT3/TSH (p=0.015). TRAb (0.99 [0.98–0.99]) and FT3/TSH (0.86 [0.84–0.88]) had the greatest AUCs. The optimal cutoff value of TRAb for GD was 0.84 IU/L (sensitivity and specificity of 98% and 99%, respectively). The optimal cutoff value of FT3/TSH for GD was 1.99 pmol/mIU (sensitivity and specificity of 79% and 80%, respectively). When the specificity of FT3/TSH for GD was set at 90%, the cutoff shifted to 3.6 pmol/mIU; at this cutoff, the sensitivity for GD was reduced to 59.6%.

**Table 3:** Receiver operating characteristic curve analysis of thyroid-related parameters for distinguishing Graves’ disease from autoimmune thyroiditis.

Parameters	AUCs95% CI	*p*	Cutoff	Sensitivity	Specificity
TT4 (ug/dl)	0.69 (0.66–0.72)	<0.0001	15.10	0.66	0.66
TT3 (ng/dl)	0.78 (0.74–0.81)	<0.0001	265.05	0.69	0.80
FT4 (pmol/L)	0.74 (0.70–0.77)	<0.0001	42.96	0.59	0.8
FT3 (pmol/L)	0.78 (0.75–0.82)	<0.0001	13.05	0.72	0.78
TSH (uIU/ml)	0.81 (0.79–0.84)	<0.0001	0.006	0.71	0.75
TT3/TT4 (ng/ug)	0.78 (0.75–0.81)	<0.0001	16.71	0.73	0.75
FT3/FT4	0.71 (0.67–0.74)	<0.0001	0.39	0.65	0.74
TT4/TSH (ug/uIU)	0.82 (0.79–0.85)	<0.0001	23.12	0.75	0.74
TT3/TSH (ug/uIU)	0.85 (0.83–0.88)	<0.0001	0.40	0.76	0.81
FT4/TSH (pmol/mIU)	0.83 (0.81–0.86)	<0.0001	5.99	0.76	0.75
FT3/TSH^#^a (pmol/mIU)	0.86 (0.84–0.88)	<0.0001	1.99	0.79	0.80
TRAb* (IU/L)	0.99 (0.98–0.99)	<0.0001	0.84	0.98	0.99
TPO-Ab (IU/ml)	0.63 (0.59–0.66)	<0.0001	45.20	0.78	0.51

*area under receiver operating characteristic curve (AUC) compared with those of TSH, TT4/TSH, TT3/TSH, FT3/TSH, and FT4/TSH, P<0.0001; ^#^AUC compared with those of FT4/TSH, TT4/TSH, and TSH, P<0.001; a: AUC compared with those of TT3/TSH, P=0.015.

TRAb, TSH receptor antibody; TT3, total triiodothyronine; TT4, total thyroxine; FT3, free triiodothyronine; FT4, free thyroxine; TSH, thyroid-stimulating hormone; TPO-Ab, thyroid peroxidase antibody; CI, confidence interval.

Of 650 patients with GD, 15 patients had TRAb levels below the cutoff; 12 of those 15 patients had FT3/TSH ratios above the cutoff, while three of the 15 patients had FT3/TSH ratios below the cutoff. The three patients with low FT3/TSH ratios were diagnosed with GD on the basis of elevated Tc-99m uptake. Of 155 patients with AIT, only three patients had TRAb levels above the cutoff; however, those three patients had FT3/TSH ratios below the cutoff and exhibited reduced Tc-99m uptake.

### Validation of FT3/TSH Ratio for Distinguishing GD From AIT Among Patients in Cohort 2

We tested the effectiveness of FT3/TSH for distinguishing GD from AIT among patients in cohort 2. Of 14 patients with AIT, all had FT3/TSH ratios below the cutoff; three of those 14 patients had TRAb levels above the cutoff, but had reduced Tc-99m uptake. Of 133 patients with GD, 25 had FT3/TSH ratio below the cutoff, while their TRAb levels were above the cutoff. Among patients in cohort 2, for the FT3/TSH cutoff of 1.99 pmol/mIU, the sensitivity and specificity for GD were 81.2% and 100%.

## Discussion

To the best of our knowledge, this is the first study to report the FT3/TSH ratio as an effective index to distinguish GD from AIT in two independent cohort. The optimal cutoff for the FT3/TSH ratio was 1.99 pmol/mIU (79% sensitivity and 80% specificity). We observed that thyrotoxicosis was more severe in patients with GD than in patients with AIT. Our results indicated that TRAb level was the optimal parameter for distinguishing patients with GD from those with AIT, among all the thyroid-related parameters (excluding RAI and technetium-99m uptake). The optimal TRAb cutoff was 0.84 IU/L, which exhibited the highest sensitivity (98%) and specificity (99%). Because GD and AIT are two of the most frequent thyrotoxicosis etiology in patients with autoimmune thyroid disease, combining TRAb level and FT3/TSH ratio might help to distinguish GD from AIT in clinical settings where RAI and Tc-99m uptake analyses are unavailable or contraindicated, especially among patients without typical clinical manifestations of GD. When inconsistent TRAb and FT3/TSH results are observed at diagnosis, RAI and Tc-99m uptake assessments should be ordered. When these tests are contraindicated, close follow-up is needed.

The stimulating activity of TRAb is the main contributor to GD onset and maintenance by means of TSH-mimicking action, which explains why TRAB level is ideal for differential diagnosis of GD. However, the TRAB cutoff for distinguishing GD from AIT has not been well established. This parameter differs according to the severity of thyrotoxicosis, duration of thyrotoxicosis, size of study sample, type of thyroid disease, and method of TRAb measurement (19–27). In one study that used an assay method similar to ours, based on ROC analysis of patients with newly diagnosed GD, patients without GD (i.e., patients with thyroid-related diseases such as AIT, subacute thyroiditis, thyroid cancer, and non-autoimmune goiter), and normal controls, the optimal TRAb cutoff was 1.75IU/L (99% sensitivity and 99% specificity) (23). In our study with larger numbers of patients who had newly diagnosed GD and AIT, the optimal cutoff was o.84 IU/L; this cutoff is presumably more appropriate for differential diagnosis of thyrotoxicosis etiology in clinical practice because it was established based on the analysis of the two main types of thyrotoxicosis (GD and AIT), instead of a composite group of patients with various non-autoimmune thyroid disease. The inclusion of patients with non-autoimmune thyroid disease might influence the cutoff determination due to differences in pathogenesis among these disease. Indeed, we found that 42 of 650 patients with GD in cohort 1 and 13 of 133 patients with GD in cohort 2 had TRAb levels below 1.75 IU/L. Thus, if GD were diagnosed using a cutoff of 1.75 IU/L, 7% of patients with GD would be missed; proper treatment to these patients would be delayed.

In 1978, a small study showed that the TT3/TT4 ratio could be used to distinguish thyroid destruction-induced thyrotoxicosis from GD. In that study, all patients with GD (n=17) had TT3/TT4 ratios above 20 ng/μg; all patients with subacute thyroiditis (n=7) and most patients with AIT (n=11) had TT3/TT4 ratios below 20 ng/μg (10). Unfortunately, this ratio was not thoroughly evaluated in a larger study, although the American Thyroid Association recommended that it could be used for differential diagnosis of thyrotoxicosis (9). In our study, ROC analysis of the TT3/TT4 ratio showed that its AUC was 0.78(95% confidence interval: 0.75-0.81), which was lower than the AUCs of the TRAb level and FT3/TSH ratio (**Table 3**). The optimal cutoff was 16.7 ng/μg, but its sensitivity(73%) and specificity(75%) were inadequate for use in clinical practice. When the cutoff was set at 20 ng/μg, the specificity increased to 90%, although the sensitivity decreased to 46%; this ratio thus might provide additional evidence to support the diagnosis of GD, at the expense of sensitivity.

It has been suggested that the limitations of the TT3/TT4 ratio in differential diagnosis of thyrotoxicosis might be related to the thyroxine-binding globin concentration, thyrotoxicosis complications, and low T3 syndrome (13). Thus, the FT3/FT4 ratio has been explored for possible use in the differential diagnosis of thyrotoxicosis; it remains controversial because of inconsistent results among previous studies (12, 16–19). In our study, ROC analysis of the FT3/FT4 ratio showed that its AUC was 0.71, suggesting that this ratio is not a good parameter for distinguishing GD from AIT.

We observed greater TSH suppression severe in patients with GD than in patients with AIT, consistent with previous findings (28). The TSH levels of most patients with GD, but not destructive thyroiditis, are reportedly suppressed below 0.005 mU/l (29). Subclinical GD is present for several months before the development of overt hyperthyroidism. During this period, TRAb is present in serum and the TSH concentration is declining. Thyrotoxicosis may be more rapid in patients with destructive thyroiditis than in patients with GD. Moreover, thyrotroph function recovered earlier after normalization of free thyroid hormone levels in patients with destructive thyroiditis, compared with patients who had GD, which implies a difference in the degree of TSH suppression. Because the TSH level is influenced by thyrotoxicosis severity and duration, TSH is presumably suitable as an indicator for distinguishing GD from AIT. In our study, ROC analysis of TSH showed that its AUC was 0.81, while its sensitivity and specificity were 71% and 75%, respectively. When specificity was set at 90%, the TSH cutoff was 0.0035 mU/l; however, its sensitivity was then reduced to 45%, suggesting that a lower TSH level is more likely to indicate the presence of GD.

Only 20% of circulating T3 is released from the thyroid in euthyroid individuals; this proportion increases to two-thirds in patients with hyperthyroidism (30). Moreover, because TSH suppression is more severe in patients with GD than in patients with AIT, we suspected that the FT3/TSH ratio would be greater in patients with GD than in patients with AIT. This ratio is likely to be a more robust indicator for distinguishing GD from AIT, compared with single parameters (e.g., FT3 or TSH). In our study, the FT3/FT4 ratio was higher in the GD group than in the AIT group. Notably, the AUC of the FT3/FT4 ratio was higher than the AUCs of FT3, FT4, TSH, TT3, and TT4 levels; it was also higher than the AUCs of the TT3/TSH, TT4/TSH, and FT4/TSH ratios. The optimal cutoff was 1.99 pmol/mIU (79% sensitivity and 80% specificity). When the cutoff was set at 3.6 pmol/mIU, the specificity was 90% and sensitivity was 60%. Thus, the FT3/FT4 ratio was the best non-isotope indicator, with the exception of TRAb. Furthermore, we found that the FT3/TSH ratio was especially useful for diagnosis of GD among patients who had TRAb levels within the normal range.

There were some strengths in this study. This large study was designed to investigate potential thyroid-related indicators for differential diagnosis of thyrotoxicosis. On the basis of sensitivity (0.8) or specificity (0.8), α=0.05, and permissible error (0.1), as well as the reported prevalence of GD in patients with thyrotoxicosis (0.8) (31), 234 patients with thyrotoxicosis were needed in this study. Thus, our study had a sufficiently large sample size. Because all patients were newly diagnosed and had not received any intervention measures, potential effects of drugs and comorbidities were minimized, which might have reduced biases associated with confounding characteristics.

There were several limitations in this study. First, this was a single-center retrospective study. Although we identified a new TRAb cutoff for distinguishing GD from AIT, this cutoff requires confirmation in larger, prospective, multicenter studies. Second, other clinical characteristics of patients (e.g., thyroid volume, Graves’ orbitopathy status, and smoking habits) were not assessed, which might affect our results, more detailed clinical information in the future studies were needed to improve our study. Third, cohort 2 included a very small number of patients with AIT. Because most patients in cohort 2 were collected in 2020, the impact of coronavirus disease 2019 presumably led to fewer clinic visits by patients with thyrotoxicosis, especially those with AIT who typically experience mild disease (compared with patients who have GD). Therefore, larger studies are needed to confirm our findings concerning patients with AIT. Finally, it has been reported that iodine intake is adequate among inhabitants of mainland China (32). Because we did not measure urinary iodine, we could not determine the iodine nutritional status among included patients, which might have affected the rate of RAI uptake by the thyroid. Notably, most patients with AIT were diagnosed by Tc-99m thyroid scan and uptake. Only low rates of RAI uptake by the thyroid in patients without oral intake of iodine-containing food and drugs within the previous 2–4 weeks were used for diagnosis of thyrotoxicosis due to AIT. These diagnoses were confirmed by the presence of spontaneous hypothyroidism without antithyroid drug treatment during follow-up visits. Thus, we made considerable efforts to avoid misdiagnosis.

In summary, our findings indicate that the FT3/TSH ratio is a novel and useful index for distinguishing GD from AIT. In combination with the TRAb level, this ratio can help to distinguish patients with GD from those with AIT, without the requirement for Tc-99m or RAI assessments.

## Data Availability Statement

The original contributions presented in the study are included in the article/supplementary material. Further inquiries can be directed to the corresponding authors.

## Ethics Statement

The studies involving human participants were reviewed and approved by Ethics Committee of Peking University People’s Hospital. Written informed consent for participation was not required for this study in accordance with the national legislation and the institutional requirements.

## Author Contributions

ZW, YZ, and XH performed data analysis and wrote the manuscript. LJ and and XH designed the study, contributed to the discussion, and reviewed and edited the manuscript. ZW, YZ, MZ, CW, LZ, WL, WY, SZ, and QR collected the data. All authors approved the final draft. All authors contributed to the article and approved the submitted version.

## Conflict of Interest

The authors declare that the research was conducted in the absence of any commercial or financial relationships that could be construed as a potential conflict of interest.

## Abbreviations

AIT, autoimmune thyroiditis; GD, Graves’ disease; FT3, free triiodothyronine; FT4, free thyroxine; RAI, radioactive iodine; Tg-Ab, thyroglobulin antibody; TPO-Ab, thyroid peroxidase antibody; TRAb, TSH receptor antibody; TSH, thyroid stimulating hormone; TT3, total triiodothyronine; TT4, total thyroxine.
